# Supplementary figures and images for: Lifestyle factors associated with pre-metabolic syndrome in young adults: A cross-sectional study of annual health examinations in university students
Source: PLoS One. 2026 Feb 11;21(2):e0342228. doi: 10.1371/journal.pone.0342228 (PMC12893567; doi:10.1371/journal.pone.0342228)

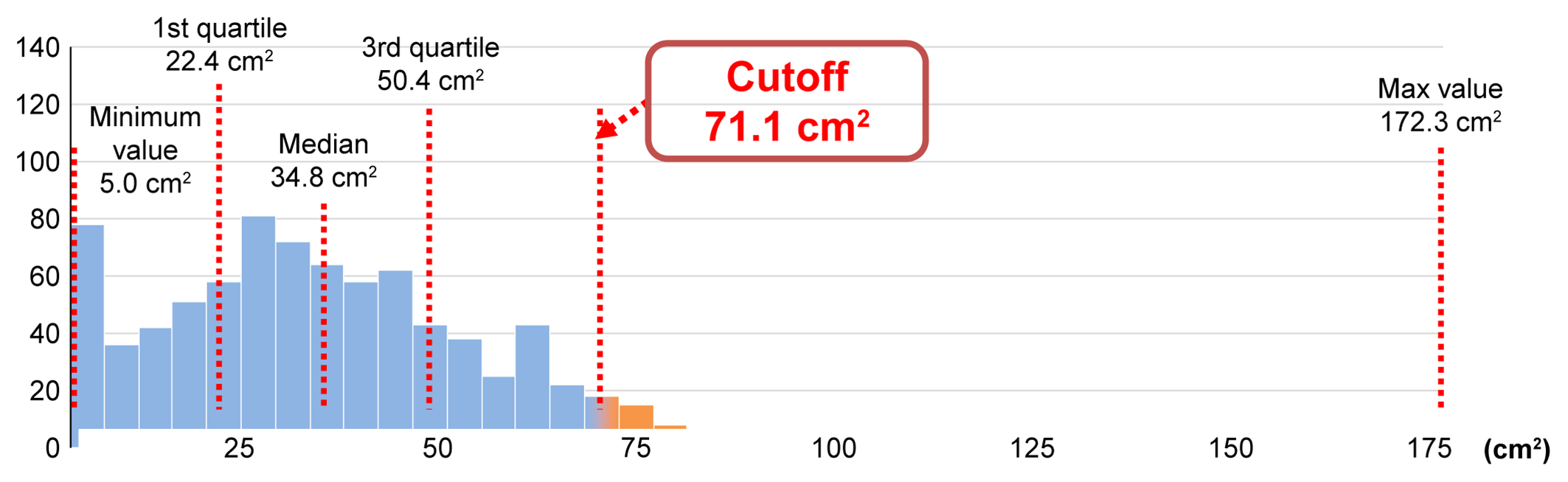

Supplement: S1 Fig — (TIF) [file pone.0342228.s001.tif]

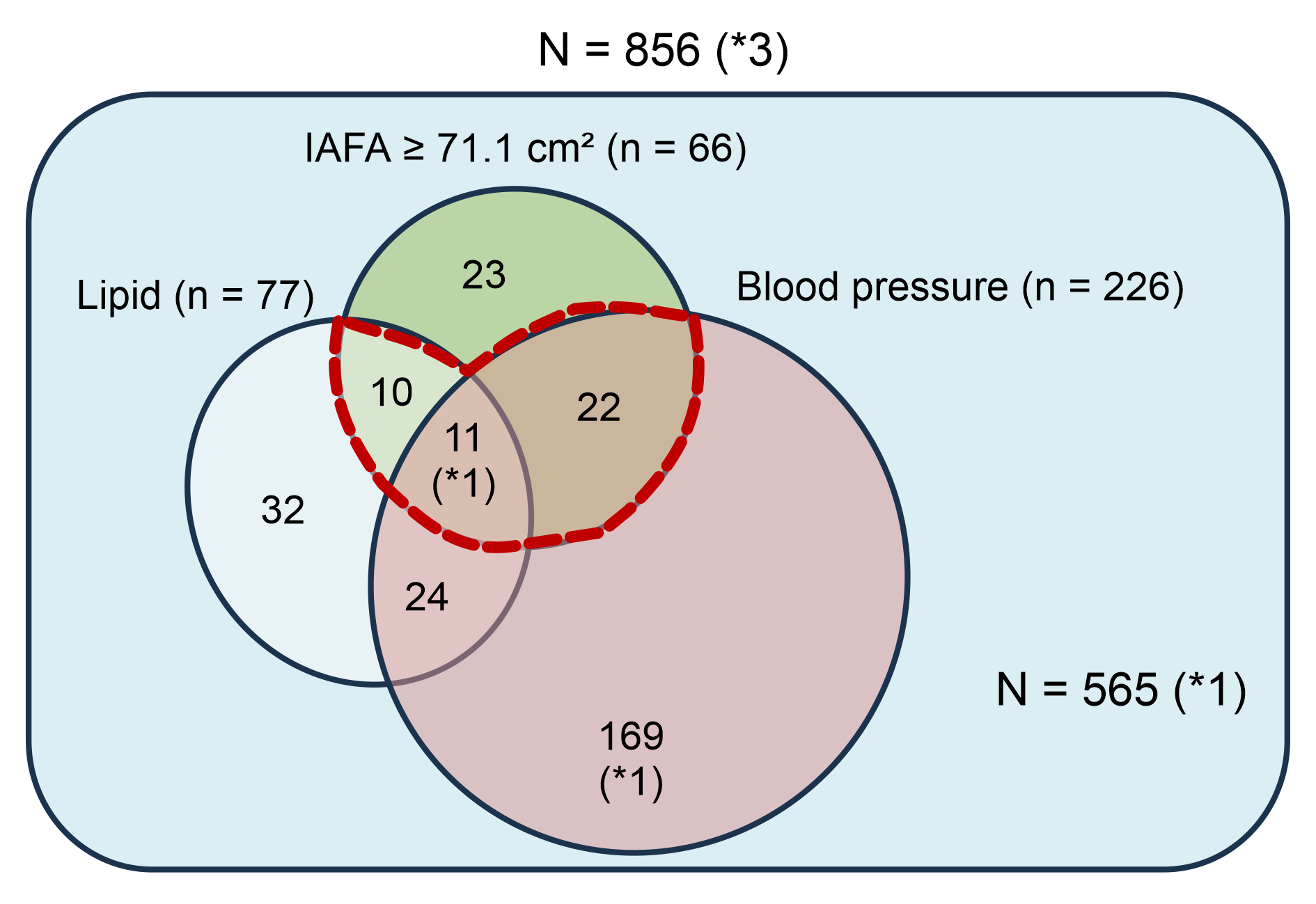

Supplement: S2 Fig — IAFA (intra-abdominal fat area), participants with IAFA ≧71.1 cm2. Blood pressure, participants with SBP ≧ 130 mmHg and/or DBP ≧ 85 mmHg. Lipid, participants with fasting TG > 150 mg/dL, non-fasting TG ≥ 175 mg/dL, and/or HDL-c < 40 mg/dL. *Includes participants with hyperglycemia (fasting plasma glucose ≥110 mg/dL or casual plasma glucose ≥200 mg/dL). The number following the asterisk indicates the number of participants with hyperglycemia. For example, N = 856 (*3) indicates that out of 856 participants, 3 had hyperglycemia. The area enclosed by red dotted lines indicates the pre-MetS group: participants with high IAFA (71.1 cm2) and at least one MetS component (i) SBP ≧ 130 mmHg and/or DBP ≧ 85 mmHg, (ii) fasting TG > 150 mg/dL, non-fasting TG ≥ 175 mg/dL, and/or HDL-c < 40 mg/dL, and/or (iii) fasting plasma glucose ≥110 mg/dL or casual plasma glucose ≥200 mg/dL). (TIF) [file pone.0342228.s002.tif]
