# Supplementary material for: Lifestyle factors associated with pre-metabolic syndrome in young adults: A cross-sectional study of annual health examinations in university students
Source: PLoS One. 2026 Feb 11;21(2):e0342228. doi: 10.1371/journal.pone.0342228 (PMC12893567; doi:10.1371/journal.pone.0342228)
Supplement: Attachment — Submitted filename: Response to Reviewer.docx [file pone.0342228.s005.docx]

Point-by-point review

**Reviewer #1:** The authors have made meaningful improvements from the first submission, especially in refining the pre-MetS definition, adjusting models for key covariates, and clarifying some results. However, the study still has serious limitations in generalizability, methodological robustness, and depth of interpretation. I would recommend major revision as the dataset is valuable, but the framing needs to be sharpened. Please address following comments in your manuscript:

**●1. The abstract is clearer than the original submission, but it still feels data-heavy. The authors list too many cutoffs and criteria, which might overwhelm general readers. The abstract would benefit from being more focused on the main findings rather than technical thresholds.**

Response 1:

Thank you for this important comment. Accordingly, we simplified the abstract by removing detailed cutoff values and focusing on the key findings (p.3, L.39).

**●2. The definition of pre-MetS has improved with the adoption of an externally validated IAFA cutoff, but it remains somewhat arbitrary. Combining IAFA ≥71.1 cm² with “at least one MetS component” is logical but not universally recognized. The authors should emphasize that this is exploratory and not a validated diagnostic construct.**

Response 2:

Thank you for pointing this out. We agree with the reviewer regarding the pre-MetS definition used in this study that it should be exploratory, since it has not been officially validated as a diagnostic criterion. For more clarification, we added sentences in both the Methods and Discussion sections (p.8, L.141-145, p.25, L.327-329)

**●3. The study population remains narrow and limits generalizability. Using only fourth-year students from one Japanese university with a 59% response rate introduces institutional and selection bias. While this is acknowledged in the limitations, the discussion still overstates the broader relevance.**

Response 3:

Thank you for this insightful comment. We agree that the generalizability of our findings is limited due to the single-institution design and specific student cohort. Accordingly, we have revised the Limitations section (p.24-25, L.312-316) to emphasize the restricted representativeness of our study population and to caution against overgeneralization.

**●4. Lifestyle data collection relies heavily on self-reporting, which undermines accuracy. Gaming time, alcohol, sleep, and diet are prone to recall and desirability bias. No objective measures (e.g., actigraphy, dietary recall validation) are included. This limitation is mentioned but downplayed.**

Response 4:

We agree with the reviewer’s comment that excessively relying on self-reported data might have undermined the accuracy of lifestyle variables. Accordingly, we have revised the *Limitations* section (p.25, L.316-318) to emphasize that all lifestyle data were heavily reliant on self-reports, subject to recall and social desirability biases, and not validated by objective measures such as actigraphy or dietary records.

**●5. The sample size of the pre-MetS group (n=43) is quite small. This raises concerns about statistical power and the stability of regression estimates, especially when multiple covariates are included. Wide confidence intervals (as seen in Tables 2–3) make some findings borderline.**

Response 5:

Thank you for your comment. We agree that the small sample size of the pre-MetS group may have affected the robustness of the regression analyses. Accordingly, we have added a new sentence in the *Limitations* section (p.25,L.325–327) to note that the small number of participants (n = 43) could have reduced the statistical power and stability of the regression estimates.

**●6. The logistic regression is now adjusted for age, sex, and BMI, which is an improvement. However, other potential confounders (family history, physical activity intensity, socioeconomic status) are not considered. Without these, the associations remain weakly interpretable.**

Response 6:

Thank you for pointing out the absence of several potential confounding factors. We agree that variables such as family history, physical activity intensity, and socioeconomic status could have influenced the results. Accordingly, we have added a sentence in the *Limitations* section (p.25, L.323-325) to note that the omission of these confounders may have limited the interpretability.

**●7. Gaming time is a repeated focus but not well contextualized. While associations are statistically significant, the mechanism is speculative and over-discussed without real behavioral data. The authors should temper claims and note that gaming may just be a proxy for sedentary behavior.**

Response 7:

We agree with the reviewer’s comment that the discussion of gaming time should be more concise and better contextualized. Accordingly, we have revised this part in the *Discussion* section (p.22-23, L.268–281) to simplify the interpretation where we clearly stated that the mechanisms linking gaming time to metabolic risk remain speculative and that gaming time may act as a proxy for sedentary behavior rather than a direct cause.

**●8. The interpretation of part-time job absence as a risk factor is still weak. The link is attributed to lower physical activity, but no actual activity measures were collected. This conclusion remains speculative and needs rephrasing as a hypothesis rather than an established link.**

Response 8:

We agree with the reviewer’s comment that the interpretation regarding the absence of a part-time job as a risk factor should be presented as a hypothesis rather than a definitive conclusion. Accordingly, we have revised the *Frequency of part-time job* in the *Discussion* section (p.23, L.286–288) and clarified that this interpretation is hypothetical and that no objective measures of physical activity were collected. We have also slightly modified the *Limitations* section to emphasize the need for future studies incorporating objective activity data.

**●9. Dietary variables are underutilized. Breakfast frequency and dining out are reported but brushed aside after regression. These findings should be better discussed, even if non-significant, since diet is a major driver of MetS risk.**

Response 9:
We agree with the reviewer’s comment that dietary variables should be discussed in more detail, even though they were not significant in the multivariable analysis. Accordingly, we have added a new paragraph in the *Discussion* section (p. 23-24, L. 290-298) to explain the potential reasons for the loss of significance after adjustment for confounders and to acknowledge that dietary habits remain important behavioral factors influencing metabolic health, consistent with previous studies.

**●10. Figures and tables have improved but remain difficult to interpret. Figure 2 (forest plot) is crowded and not reader-friendly. A simpler bar or line visualization of trends across categories might be more accessible.**

Response 10:
We thank the reviewer for this helpful comment regarding Fig. 2. Accordingly, we have replaced the original forest plot with a simplified bar graph that presents the odds ratios (ORs) for each category, accompanied by numerical annotations showing the corresponding 95% confidence intervals, *p*-values, and sample sizes. This design improves readability and allows easier visual comparison across categories.

**●11. The discussion section still restates results at length. It lacks deeper integration with broader literature, especially studies in comparable young-adult populations globally. There’s little attempt to contrast findings with non-Asian cohorts.**

Response 11:

Thank you for your suggestion. We have revised the *Discussion* section and integrated the recent findings from Western, Middle Eastern, and African university populations that similarly reported links between inactivity, sedentary behavior, unhealthy diet, and metabolic risk. This addition provides a clearer international context for our findings (p.24, L.300–309).

**●12. Public health implications are vague. The conclusion states “further prospective studies are warranted,” which is valid but generic. More concrete policy or campus-level interventions (e.g., health promotion programs targeting sedentary behavior) would make the study more meaningful.**

Response 12:

We thank the reviewer for this helpful suggestion. Therefore, we have revised the *Conclusion* section (p.26, L.338-344) and included more concrete and practice-oriented public health implications. Specifically, we mentioned campus-level health promotion initiatives, such as programs targeting sedentary behavior and promoting balanced daily routines among university students.

**●13. The manuscript acknowledges many limitations, but some are minimized. For instance, IAFA by BIA is noted as less reliable than CT/MRI, but the authors still present it as highly accurate. This balance should be more cautious.**

Response 13:

We appreciate the reviewer’s comment regarding the precision of IAFA estimation by BIA. To address this, we have revised the *Methods* section (p.6, L.106-110) and clarified that BIA provides a practical but less precise estimate of visceral fat compared with CT or MRI.

**●14. The novelty is modest. IAFA use in a student cohort is interesting, but the associations with gaming and job frequency are neither surprising nor groundbreaking. The contribution lies mainly in providing updated Japanese student data, which should be more clearly stated as its niche value.**

Response 14:

Thank you for this helpful suggestion. We agree that the novelty of our study is modest. To better clarify the study’s contribution, we have added a statement in the *Discussion* section (p.24, L.306–309) emphasizing that this research provides updated and population-specific data on intra-abdominal fat and lifestyle factors among Japanese university students, thereby offering a timely and context-specific contribution to understanding metabolic health in young adults.

**●15. Language is clearer than before, but the manuscript remains verbose. Redundancies and over-explanations (e.g., lengthy justification of known MetS pathways) could be trimmed to improve flow and readability.**

Response 15:

Thank you for your helpful comment. We agree that some sections, particularly those describing general MetS mechanisms and behavioral pathways, were verbose. We have carefully revised the *Introduction* and *Discussion* sections to remove redundant explanations, condense descriptions of well-known concepts, and improve overall readability and flow.

**Reviewer #2**: **Thank you for improving the manuscript and addressing reviewers comments. I highly appreciate research team efforts**

Response to Reviewer #2:

We sincerely thank you for the positive evaluation and kind acknowledgment of our efforts to improve the manuscript. We greatly appreciate your encouraging feedback and are pleased that the revisions have addressed your previous concerns.

**Reviewer #3**: **All comments from the previous review have been addressed. This manuscript is suitable for publication.**

Response to Reviewer #3:

We are deeply grateful for your careful review of our revised manuscript and for your positive feedback. We are pleased to be informed that all prior comments have been satisfactorily addressed and that the manuscript is now considered suitable for publication.
