# Supplementary material for: Lifestyle factors associated with pre-metabolic syndrome in young adults: A cross-sectional study of annual health examinations in university students
Source: PLoS One. 2026 Feb 11;21(2):e0342228. doi: 10.1371/journal.pone.0342228 (PMC12893567; doi:10.1371/journal.pone.0342228)
Supplement: Attachment — Submitted filename: CM_R2R.docx [file pone.0342228.s006.docx]

Response to Additional Editor Comments

We sincerely thank the Academic Editor for the constructive and helpful comments. We have revised the manuscript accordingly as detailed below.

Additional Editor Comments:

●1. Please state that the definition of IAFA ≥71.1 cm² + ≥1 MetS component remains an exploratory analysis and, therefore, should be recognized as a potential limitation in the Discussion.

Response 1:

As suggested, we have explicitly stated that the operational definition of pre-MetS (IAFA ≥71.1 cm² plus ≥1 MetS component) is based on an exploratory analysis rather than on a validated diagnostic criterion. To emphasize this point, this statement is presented as the first study limitation, clearly recognizing it as a key methodological limitation of this study (Discussion, p. 24, L. 311–314).

●2. Another potential limitation is the sample size in the pre-MetS group. With only 43 patients, the authors should recognize the lack of statistical power to support associations and acknowledge that a larger sample size would be required to adjust for potential unmeasured confounders.

Response 2:

We have acknowledged that the relatively small number of participants in the pre-MetS group (n = 43) limits statistical power and restricts adjustments for potential unmeasured confounders. To highlight its importance, this issue is described as the second limitation, emphasizing the need for larger sample sizes in future studies to support the observed associations (Discussion, p. 24–25, L. 314–317).

●3. The authors still use the term “risk factor,” which should be changed to “associated factor” or phrased as a “positive association,” as the study design cannot infer causality.

Response 3:

In accordance with the editor’s recommendation, we have carefully reviewed the manuscript and revised relevant terminologies to avoid causal interpretations. Although the terms “associated factor” and “positive association” are used sparingly where appropriate, we have consistently replaced causal language, such as “risk factor,” with expressions that emphasize non-causal relationships, including references to associations and early metabolic alterations related to MetS. These revisions have been made throughout the manuscript to emphasize the cross-sectional design (p. 3, L. 45; p. 4, L. 53 and 67–68; p. 5, L. 76; p. 6, L. 94; p. 8, L. 146; p. 10, L. 185; p. 23, L. 272 and 279-280; p. 24, L. 301; and p. 26, L. 339-342).

●4. In Figure 2, please consider using a single point estimate with 95% confidence intervals instead of bars only.

Response 4:

Following the editor’s suggestion, Figure 2 has been revised from a bar chart to a forest plot, displaying single point estimates with 95% confidence intervals. Accordingly, the figure legend has also been updated to improve clarity and consistency with the revised figure and its legend (p. 22, L. 255).
